# Complete mitochondrial genome sequence for the cuneate drum *Nibea miichthioides* (Perciformes, Sciaenidae) and its phylogeny

**DOI:** 10.1080/23802359.2016.1172037

**Published:** 2016-06-20

**Authors:** Zehui Hu, Yuebin Wang, Xuejun Chai, Dongfa Zhu

**Affiliations:** a Key Laboratory of Applied Marine Biotechnology of Ministry of Education, Ningbo University, Ningbo, PR China;; b Key Laboratory of Mariculture and Enhancement of Zhejiang Province, Marine Fisheries Research Institute of Zhejiang, Zhoushan, PR China

**Keywords:** Mitochondrial DNA, *Nibea miichthioides*, phylogenetic analysis, Sciaenidae

## Abstract

The cuneate drum, *Nibea miichthioides*, is a native sciaenid species with relevant commercial importance in China. However, the wild resource represented by this species has been severely damaged owing to overfishing and water pollution. For conserving and recovering the species through appropriate measures, genetic studies on the population are necessary. The complete mitochondrial genome of *N. miichthioides* is a supplement to the mitogenome database for giant croakers and can be used to address taxonomic problems and phylogenetic relationships in Sciaenidae. In this study, we sequenced and characterized the complete mitochondrial genome of *N. miichthioides*. The full length of the complete mitochondrial DNA was 16,490 bp. It contained 13 protein-coding genes, 22 tRNA genes, two rRNA genes and a control region. All 21 tRNA genes can fold into a typical cloverleaf structure except for *tRNA^Ser^* (AGY), which lacks a dihydrouridine arm. The phylogenetic analysis using the complete mitochondrial genome revealed that *N. miichthioides*, *A. amoyensis*, *N. japonica* and *A. japonicus* might be grouped in *Argyrosomus*, but not belonged to *Nibea* of Argyrosominae, which was highly consistent with the proposal of Talwar. This investigation provides an effective molecular tool for genetic research on and protection of this endangered species.

The cuneate drum, *Nibea miichthioides*, a large-sized benthonic giant croaker endemic to China, is distributed in the middle south East China Sea and South China Sea (Zhu et al. 1963; Cheng & Zheng 1987). Historically, *N. miichthioides* is a native sciaenid species with relevant commercial importance in China. The wild resource was severely damaged owing to overfishing and water pollution in the last decade (Hu et al. 2007). For conserving and recovering the species through appropriate measures, genetic studies on the population are necessary. In this study, we sequenced and characterized the complete mitochondrial genome to provide effective molecular tools for the genetic research of *N. miichthioides*. Presently, phylogenetic relationship of the Sciaenidae was inconsistent with different researches. *Argyrosomus* and *Nibea* should be classified into Argyrosominae (Zhu et al. 1963), while Chen (2007) found that *N. miichthioides* and *Argyrosomus* did not cluster with other *Nibea* species using 16S rRNA gene. In addition, Talwar (1995) proposed that *N. miithchioides* and *N. japonica* should be renamed *A. amoyensis* and *A. japonicus*, which have closer relationship with Otolithinae. Researches of single gene or taxonomic might lose some significant evolutionary characters. Hence, this study expect to contribute to the phyogenetic analysis of the Sciaenidae and natural resources conservation of *N.miithchioides*.

In this study, the specimen of *N. miichthioides* sampled from Ningde city, Fujian, South China Sea was stored in the fish collection of Zhejiang Marine Fisheries Research Institute. The caudal fins of *N. miichthioides* were scissored for genomic DNA isolation. Total genomic DNA was isolated using the high-salt procedure (Aljanabi & Martinez 1997). PCR primers were initially designed according to *N. albiflora* (HQ890947.1), *Sciaenops ocellatus* (JQ286004.1) and *Miichthys miiuy* (HM447240.1). Subsequently, based on the received sequences, some additional primers were designed to supplement residual gaps. Finally, ContigExpress software was used for sequence analysis and assembly. The assembled mitochondrial genome was annotated with MitoFish (Iwasaki et al. 2013). All tRNA genes were reappraised by tRNAscan-SE1.21 (Lowe & Eddy 1997), which was also used to characterize the anti-codons of all tRNAs. Simultaneously, we downloaded 18 complete mitochondrial genome of the Sciaenidae, which were aligned by means of Clustal W using BioEdit (Hall 2004). The best-fit model to nucleotide substitution of these genomes were Jmodel test2 (Darriba et al. 2012), via Alkaike information criteria (AICc). Finally, the phylogenetic analysis of Maximum Likelihood(ML) was performed using MEGA 5.0 (Tamura et al. 2011), and the number of bootstrap replicates is 1000.

The complete mitochondrial genome of *N. miichthioides* was 16,490 bp in length (KU738606). It contained 22 tRNA genes, 13 protein-coding genes, two rRNA genes and a control region. All genes were encoded on the heavy strand except *ND6* and eight tRNAs. The length of all tRNAs ranged from 66 to 74 bp and their anti-codons were consistent with other fish of Sciaenidae. All 21 tRNA genes can fold into a typical cloverleaf structure except for *tRNA^Ser^* (AGY) which lack a dihydrouridine arm. Usually, the function of tRNAs is mainly determined by anticodon stem-loop and amino acid stem. Hence, the lack of dihydrouridine arm does not affect the function (Hanada et al. 2000). All the 13 protein-coding genes were initiated with the orthodox ATG. They had three types of intact stop codons (TAG, AGA and TAA) and two types of incomplete stop codons (TA– and T–). The two rRNA genes (12S rRNA and 16S rRNA) were typically isolated by *tRNA^Val^*, located between *tRNA^Phe^* and *tRNA^Leu^*. The control region is located between the *tRNA^Phe^* and *tRNA^Thr^* genes on the heavy strand with the size of 824 bp. Some intergenic nucleotides (from - 10 to 36) were also recognized. The mitogenome base composition was 27.0% for A, 25.3% for T, 31.0% for C and 16.7% for G. The A + T content (52.3%) was higher than the G + C content.

The best-fit model to nucleotide substitution of these genomes was HKY + G + I. Phylogenetic analysis revealed that *N. miichthioides*, *A. amoyensis* and other eight fish first clustered into the Argyrosominae clade (Figure 1). Then, the Argyrosominae, Pseudosciaeninae and Sciaeniae formed the sister group, while the Johniinae became a separate clade, which is accordant with the researches (Chen 2007). In the present study, the phylogenetic analysis showed that *N. miichthioides*, *A. amoyensis*, *N. japonica* and *A. japonicus* were clustered into sister group (Figure 1), being inconsistent with the previous reports (Zhu et al. 1963; Cheng et al. 1987). Due to high bootstrap values’ support, phylogeny validated that *N. miichthioides*, *A. amoyensis*, *N. japonica* and *A. japonicus* might be grouped in *Argyrosomus*, but not belonged to *Nibea* of Argyrosominae, which was highly consistent with the proposal of Talwar (1995).

**Figure 1.**
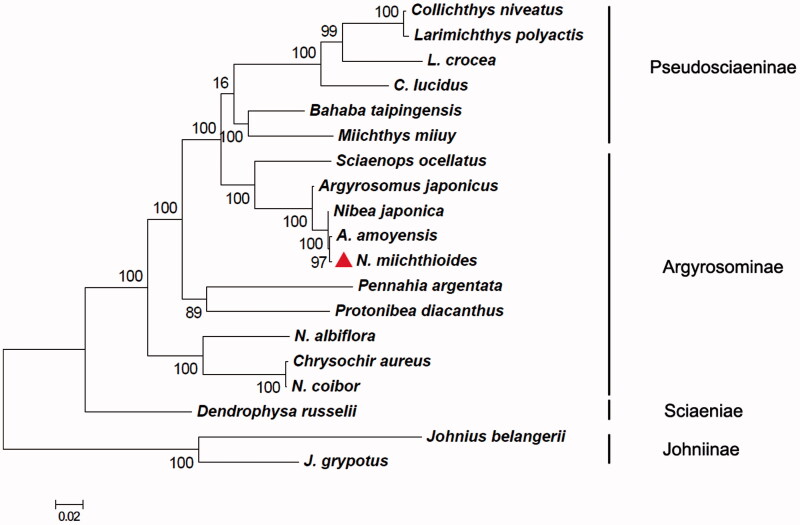
The phylogenetic relationship for fish of Sciaenidae. *Note*: 0.02 is the evolution scale of phylogenetic tree. GenBank Accession: *Argyrosomus japonicus* (JQ728563), *A. amoyensis* (Argyrosomus amoyensis, KM257863), *Bahaba taipingensis* (JX232404), *Chrysochir aureus* (JQ692068), *Collichthys niveatus* (HM219223), *C. lucidus* (*Collichthys lucidus*, JN857362), *Dendrophysa russelii* (JQ728562), *Johnius belangerii* (KF211426), *J. grypotus* (*Johnius grypotus*, KC491206), *Larimichthys polyactis* (GU586227), *L. crocea* (*Larimichthys crocea*, EU339149), *Miichthys miiuy* (HM447240), *Nibea miichthioides* (KU738606, in this study), *N. japonica* (*Nibea japonica*, KT184692), *N. albiflora* (*Nibea albiflora*, HQ890947), *N. coibor* (*Nibea coibor*, KM233452), *Pennahia argentata* (KC545800), *Protonibea diacanthus* (KM257722) and *Sciaenops ocellatus* (JQ286004).
